# Thrombus Metabolism‐Based Molecular Subtyping for Prognostic Risk Stratification in Acute Ischemic Stroke: A Preliminary Study

**DOI:** 10.1002/cns.71116

**Published:** 2026-08-30

**Authors:** Taoyuan Lu, Wei Li, Wenbo Cao, Yiying Liu, Jinzi Wei, Dechao Wang, Liqun Jiao, Xin Xu

**Affiliations:** ^1^ Department of Neurosurgery Xuanwu Hospital, Capital Medical University Beijing China; ^2^ China International Neuroscience Institute (China‐INI) Beijing China; ^3^ Xuanwu Hospital Jinan Branch Jinan China; ^4^ Department of Rehabilitation Medicine The First Affiliated Hospital of Zhengzhou University Zhengzhou China; ^5^ Department of Interventional Neuroradiology Xuanwu Hospital, Capital Medical University Beijing China

**Keywords:** acute ischemic stroke, metabolomic analysis, molecular subtype, prognosis, thrombus

## Abstract

**Aims:**

To preliminarily characterize metabolic molecular subtypes of cerebral thromboemboli and evaluate their clinical significance in anterior circulation acute ischemic stroke due to large vessel occlusion (AIS‐LVO).

**Methods:**

Untargeted metabolomics was performed on thromboemboli retrieved from 36 patients with anterior circulation AIS‐LVO using ultra‐performance coupled liquid chromatography with quadrupole time‐of‐flight mass spectrometry (UPLC‐Q‐TOF‐MS). Unsupervised hierarchical clustering was employed to identify distinct metabolic molecular subtypes, and their associations with stroke etiology, radiographic severity, and functional outcomes were analyzed.

**Results:**

Two distinct thrombus metabolic molecular subtypes (C1 and C2) were identified based on 12 metabolites significantly associated with both short‐term (7‐day ∆NIHSS) and long‐term (90‐day mRS) functional outcomes. The C1 subtype, predominantly cardioembolic, exhibited enhanced lipid metabolism, whereas the C2 subtype, primarily atherothrombotic, demonstrated increased folate metabolism. Patients with C1 thromboemboli presented more severe admission ischemic lesions (as indicated by ASPECTS) and experienced poorer short‐term and long‐term outcomes. A six‐metabolite signature derived from LASSO regression was identified for exploratory discrimination of thrombus metabolic subtypes, etiological subtypes, and 90‐day outcomes.

**Conclusion:**

This preliminary exploratory study identifies two metabolically distinct thrombus molecular subtypes with clinical implications in anterior circulation AIS‐LVO, providing a novel basis for risk stratification and personalized secondary prevention and warrants further investigation.

## Graphical abstract

This study identified two metabolically distinct thrombus molecular subtypes in AIS: a lipid metabolism‐enriched subtype (C1) associated with cardioembolic stroke and poor functional outcomes, and a folate metabolism‐enriched subtype (C2) linked to atherothrombotic stroke and better prognosis. A six‐metabolite signature further demonstrated exploratory potential for discriminating thrombus metabolic molecular subtypes, etiological subtypes, and 90‐day functional outcomes.

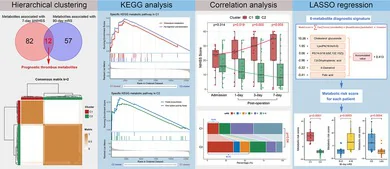

## 1 Introduction

Acute ischemic stroke due to large vessel occlusion (AIS‐LVO) is a devastating neurovascular disease with high morbidity, mortality, and recurrence rates [1]. AIS‐LVO comprises heterogeneous etiological subtypes, including cardioembolic (CE) and large artery atherothromboembolic (LAA) strokes, which differ in pathogenesis, risk profiles, severity, and outcomes, necessitating tailored adjunctive therapies and accurate etiological identification [2, 3]. Endovascular thrombectomy (EVT) has revolutionized treatment paradigms for AIS‐LVO, achieving recanalization rates exceeding 90% [4, 5]. However, approximately half of successfully recanalized patients fail to achieve functional independence, a phenomenon known as futile recanalization [6]. Consequently, significant research efforts focus on identifying prognostic factors, encompassing clinical characteristics, radiological features, and laboratory biomarkers, to refine patient selection, yet none have consistently proven reliable for clinical application [7, 8].

Metabolites, including lipids, amino acids, carbohydrates, and nucleotides, are bioactive metabolic intermediates or end‐products that integrate genotype, environment, and phenotype, rendering them promising biomarkers for diagnostic, prognostic, and disease classification [9]. Metabolic disturbances, characterized by alterations in metabolic pathways and consequent accumulation or deficiency of specific metabolites, have been mechanistically linked to AIS pathogenesis [10, 11]. Numerous serum/plasma metabolomics studies have explored AIS pathophysiology and identified potential diagnostic and prognostic biomarkers [10, 11, 12, 13, 14, 15]. However, the relevance of systemic metabolic changes to local vascular pathology remains unclear, and the clinical implications of thrombus‐specific metabolic profiles have yet to be elucidated. Our prior thrombus metabolomics study correlated thrombus metabolic profiles with stroke etiology, identifying six metabolites that distinguish CE from LAA thromboemboli [16]. Nevertheless, research on thrombus‐specific metabolic molecular subtypes and their clinical relevance, particularly regarding prognosis, remains limited in patients with AIS‐LVO. To date, only one study has proposed metabolites such as sorbitol and glucose as potential predictors of clinical outcomes [17]. Given the inherent heterogeneity of both stroke pathogenesis and patient populations, individual metabolites are unlikely to achieve sufficient predictive accuracy for clinical application [3, 7, 8]. Accordingly, this exploratory study aimed to conduct unsupervised hierarchical clustering analysis to primarily characterize distinct thrombus metabolic molecular subtypes, and explore their relationships with stroke severity, etiology, and short‐term and long‐term functional outcomes in patients with anterior circulation AIS‐LVO.

## 2 Materials and Methods

### 2.1 Study Design and Participants

We conducted a retrospective analysis utilizing prospectively maintained clinical databases and biobanks. Consecutive patients with anterior circulation AIS‐LVO who underwent successful EVT were screened from March 2023 to August 2023 at our comprehensive stroke center. Inclusion criteria were: (1) age ≥ 18 years; (2) first‐ever anterior circulation AIS‐LVO due to LAA or CE following the Trial of Org 10,172 in Acute Stroke Treatment (TOAST) classification [18]; (3) successful angiographic recanalization achieved by EVT with or without intravenous thrombolysis (IVT), as determined by the modified Thrombolysis in Cerebral Infarction score (mTICI) of 2b‐3; and (4) a premorbid modified Rankin Scale (mRS) score of ≤ 1. Exclusion criteria included: (1) severe cardiac, hepatic or renal failure; (2) recent (< 4 weeks) or active infections; (3) cancer‐related stroke [19, 20]; and (4) unavailable thrombus samples, incomplete clinical data, or missing follow‐up data.

### 2.2 Clinical Data Collection

We collected data on: (1) age and sex; (2) cardiovascular risk factors; (3) stroke etiology ultimately determined at discharge, including cardiac disorders responsible for CE stroke [21]. The etiological work‐up was based on clinical features, laboratory tests, and perioperative ancillary diagnostic studies. Patients presenting with potential coexisting CE and LAA stroke etiologies (e.g., concurrent atrial fibrillation and ipsilateral carotid stenosis ≥ 50%) were categorized as having embolic stroke of undetermined source per TOAST criteria [18], and were thus excluded from the study; (4) interventional parameters including number of passes, time metrics, and final mTICI score; (5) ischemic lesions assessed using the Alberta Stroke Program Early CT Score (ASPECTS); (6) stroke severity assessed using the National Institute of Health Stroke Scale (NIHSS). Short‐term functional outcomes were evaluated by measuring the change in NIHSS from admission to Day 7 post‐thrombectomy (7‐day ∆NIHSS); (7) no‐reflow phenomenon defined as a radiological infarct area exhibiting > 15% interhemispheric asymmetry in cerebral blood volume or flow, based on 24‐h follow‐up computed tomography perfusion imaging analyzed with RAPID software (iSchema View, version, 5.0.2) [22]; (8) symptomatic intracranial hemorrhage (sICH) defined as an increase in the NIHSS of ≥ 4 points due to intracranial hemorrhage within 24 to 36 h post‐thrombectomy [23]; and (9) long‐term functional outcomes assessed using the 90‐day mRS, with favorable and unfavorable outcomes defined as mRS scores of 0–2 and 3–6, respectively. All assessments were conducted by certified neurologists and radiologists who were blinded to the clinical data.

### 2.3 Thrombus Sample Preparation and Untargeted Metabolomic Analysis

Freshly retrieved thromboemboli were snap‐frozen in liquid nitrogen and stored at −80°C until analysis. In cases of fragmented thrombi, all fragments were pooled and considered as a single thrombus [24]. Fifty milligrams of thrombus material were homogenized in 0.5 mL of a 50% methanol/50% water solution, and supernatants were collected after centrifugation (12,000 g for 20 min at 4°C) for untargeted metabolomic analysis using ultra‐performance coupled liquid chromatography with quadrupole time‐of‐flight mass spectrometry (UPLC‐QTOF‐MS; Xevo G2‐XS QTOF, Waters) [16]. Chromatographic separation was performed on an ACQUITY UPLC BEH Amide column (1.7 μm, 2.1 × 150 mm) at 45°C with a VanGuard pre‐column (1.7 μm, 2.1 × 5 mm). The mobile phase consisted of (A) water with 0.1% formic acid and (B) acetonitrile with 0.1% formic acid. The gradient elution program was as follows: 0–2 min, 1% B (0.4 mL/min); 2–8 min, 1% to 55% B (0.4 mL/min); 8–9 min 55% to 99% B (0.4 mL/min); 9–11 min, 99% B (0.8 mL/min); 11–19 min at 1% B (0.8 mL/min), and 19–23 min, 1% B (0.4 mL/min). The injection volume was 5 μL. MS was operated in both positive and negative electrospray ionization modes. In each acquisition cycle, low‐energy and high‐energy data were collected simultaneously. The optimized MS parameters were: capillary voltage 3.5 kV, sampling cone 40 V, source offset 50 V, source/desolvation temperatures 120/550°C, cone gas (N_2_) 50 L/h, desolvation gas (N_2_) 1000 L/h, StepWave offset 10 V, MS mode sensitivity, mass range, *m/z* 50–1200, low collision energy 4 eV, and high collision energy ramp 15–45 eV. Leucine enkephalin (200 pg/μL and 5 μL/min) served as the lock‐mass for real‐time mass calibration. Data acquisition was controlled by MassLynx V4.2 (Waters). To ensure data quality and system stability, a pooled quality control (QC) sample was prepared by combining equal aliquots from all study samples and was injected at the beginning, after every 10 study samples, and at the end of the sequence. Features with a coefficient of variation (CV) > 30% across QC injections were excluded from downstream analysis.

Data processing was conducted using Waters Progenesis QI v2.0 software. Metabolite annotation was performed against the Human Metabolome Database, LipidMaps, and MassBank, with an accurate mass tolerance of ±5 ppm. The identification confidence levels were assigned according to the Metabolomics Standards Initiative (MSI) criteria [25]. Metabolites that were not quantifiable in > 80% of samples were excluded, and thrombotic‐specific differentially expressed metabolites (DEMs) were identified using the partial least squares discriminant analysis (PLS‐DA) and Welch's *t*‐test. Metabolites with a variable importance in the projection (VIP) > 1 in the PLS‐DA model, a *p* < 0.05 in Welch's *t*‐test, and an absolute log_2_ fold change (|logFC|) > 0.58 were defined as DEMs. Given the small cohort size, *p*‐value adjustment for multiple testing was not applied to avoid over‐stringency and potential loss of biologically relevant signals. Metabolic pathway enrichment analysis was performed using MetaboAnalyst v6.0 (https://www.metaboanalyst.ca/MetaboAnalyst/).

### 2.4 Unsupervised Hierarchical Clustering

To elucidate the heterogeneity of thrombus metabolism, we conducted an unsupervised hierarchical clustering using the ‘ConsensusClusterPlus’ R package, with parameters set to 100 iterations and 0.8 resampling [26]. The clustering was based on the normalized expression profiles of thrombus metabolites concurrently associated with both 7‐day ∆NIHSS and 90‐day functional outcomes (dichotomized as mRS 0–2 vs. 3–6). We identified the optimal number of clusters by analyzing the consensus score matrix and the cumulative density function (CDF) curve [27]. The heatmap of the consensus score matrix and the metabolite expression matrix of clusters were visualized using the “ComplexHeatmap” R package.

### 2.5 Metabolic Enrichment Analysis

To characterize thrombus subtype‐specific biological signatures, we developed a metabolite set enrichment analysis (MSEA) by adapting the Gene Set Enrichment Analysis (GSEA) framework [28]. All detected metabolites were ranked based on logFC values, followed by MSEA execution using the “clusterProfiler” R package. Kyoto Encyclopedia of Genes and Genomes (KEGG) pathway metabolite sets were retrieved using the “massdatabase” R package. We set the number of permutations to 10,000 to obtain normalized enrichment scores (NES), considering metabolite sets with false discovery rate (FDR) < 0.05 as significantly enriched.

### 2.6 Statistics Analysis

All data processing, statistical analyses, and graphical visualizations were conducted in R v4.3.1. Quantitative variables were presented as mean (standard deviation; SD) or median (inter quartile range; IQR), depending on the results of the Shapiro–Wilk test for normality. For comparisons between two independent groups, parametric data were analyzed using the independent Student's *t*‐test, while non‐parametric data were assessed using the Wilcoxon rank‐sum test. Longitudinal changes in NIHSS scores were evaluated using the Wilcoxon signed‐rank test. For multiple comparisons, *p‐*values were adjusted using the Benjamini–Hochberg FDR correction. Categorical variables were presented as frequencies (percentages), with intergroup differences analyzed using either the Chi‐square test or Fisher's exact test, as appropriate. Correlations between thrombus metabolites and the 7‐day ΔNIHSS were assessed using the Spearman correlation analysis. To identify key metabolites and develop a metabolite‐based diagnostic signature, Least Absolute Shrinkage and Selection Operator (LASSO) regression was employed. The model's diagnostic and predictive capabilities were assessed using receiver operating characteristic (ROC) curve analysis, with the area under curve (AUC) calculated, via the ‘pROC’ R package. Further nested 5 × 4‐fold cross‐validation, bootstrapping, permutation testing, and calibration assessment were performed to evaluate potential over‐fitting risk of the signature. Statistical significance was defined as a two‐tailed *p* < 0.05.

## 3 Results

### 3.1 Baseline Characteristics

This study included 36 patients with anterior circulation AIS‐LVO (mean age 64 ± 11 years; 41.7% female) who achieved successful angiographic recanalization and had available thrombus samples. The detailed baseline demographic and clinical characteristics are summarized in Table 1. Stroke etiologies were categorized as LAA in 16 (44.4%) patients and CE in 20 (55.6%) patients. Among the CE cases, the underlying cardiac disorders comprised atrial fibrillation (*n* = 16), patent foramen ovale (*n* = 2), rheumatic heart disease (*n* = 1), and endocarditis (*n* = 1) (Table S1). The median admission NIHSS and ASPECTS scores were 16 (IQR, 13–21.3) and 7.5 (IQR, 5–9), respectively. One patient experienced sICH post‐EVT. Despite successful macrovascular recanalization, the no‐reflow phenomenon was observed in 17 patients (47.2%). The 7‐day ΔNIHSS was −1 (IQR, −7.3–4.8), with 19 (52.8%) patients exhibiting neurological improvements (negative ΔNIHSS) and 17 (47.2%) showing a positive value or zero. A favorable 90‐day functional outcome (mRS of 0–2) was observed in 15 (41.7%) patients.

**TABLE 1:** Baseline characteristics and outcomes of the study population.

Characteristic	AIS patients (*n* = 36)	Thrombus metabolic molecular subtypes		
		C1 (*n* = 13)	C2 (*n* = 23)	*p*
Age, year, mean (SD)	64 (11)	62.5 (11.5)	65 (10.9)	0.542^a^
Gender, male/female, *n*	21/15	7/6	14/9	0.953^b^
Risk factors, *n* (%)				
Hypertension	24 (66.7)	10 (76.9)	14 (60.9)	0.468^b^
Diabetes mellitus	12 (33.3)	6 (46.2)	6 (26.1)	0.281^b^
Dyslipidemia	3 (8.3)	3 (23.1)	0 (0)	0.04^b^
Coronary artery disease	14 (38.9)	6 (46.2)	8 (34.8)	0.752^b^
Smoking	8 (22.2)	4 (30.8)	4 (17.3)	0.422^b^
Drinking	8 (2.2)	3 (23.1)	5 (21.7)	1.00^b^
Admission SBP, mmHg, mean (SD)	160.4 (20.5)	159.6 (13.8)	160.9 (23.7)	0.837^a^
Admission DBP, mmHg, mean (SD)	90.4 (13.7)	96.4 (10.4)	87 (14.3)	0.032^a^
Admission NIHSS, median (IQR)	16 (13–21.3)	24 (15–27)	16 (12–24.5)	0.314^c^
Admission ASPECTS, median (IQR)	7.5 (5–9)	5 (3–7)	9 (7–9)	0.003^c^
Stroke etiology, *n* (%)				
CE	20 (55.6)	11 (84.6)	9 (39.1)	0.022^b^
LAA	16 (44.4)	2 (15.4)	14 (60.9)	
Bridging IVT, *n* (%)	14 (38.9)	4 (30.8)	10 (43.5)	0.693^b^
Procedure parameters				
OTD, min, median (IQR)	226.5 (167.5–352)	242.0 (189–336)	225 (164–377.5)	0.432^c^
OTP, min, median (IQR)	389 (274.8–585)	286 (230–355)	290 (234.5–415.5)	0.26^c^
PTR, min, median (IQR)	108.5 (84.5–126.2)	100 (86–122)	119 (77–136.5)	0.178^c^
OTR, min, median (IQR)	430 (325, 521)	409 (322, 458)	438 (339.5, 518)	0.401^c^
Number of passes, median (IQR)	1 (1–3)	1 (1–3)	1 (1–2.5)	0.884^c^
Outcomes				
No‐reflow *n* (%)	17 (47.2)	8 (61.5)	9 (39.1)	0.299^b^
sICH, *n* (%)	1 (2.8)	0 (0)	1 (4.3)	1.00^b^
1‐day NIHSS, median (IQR)	17 (8.8–26.3)	26 (21–34)	12 (7.5–21)	0.021^c^
3‐day NIHSS, median (IQR)	15.5 (5.8–30)	30 (20–36)	10 (5–18)	0.003^c^
7‐day NIHSS, median (IQR)	15 (3–34.3)	35 (20–38)	8 (2.5–17.5)	0.002^c^
7‐day ∆NIHSS, median (IQR)	−1 (−7.3–4.8)	8 (−3–12)	−5 (−11–0)	0.002^c^
90‐day mRS of 0–2, *n* (%)	15 (41.7)	2 (15.4)	13 (56.5)	0.04^b^

Abbreviations: 7‐day ∆NIHSS, the change in NIHSS from admission to 7‐day after thrombectomy; AIS, acute ischemic stroke; ASPECTS, Alberta Stroke Program Early CT Score; DBP, diastolic blood pressure; IQR, inter quartile range; IVT, intravenous thrombolysis; mRS, modified Rankin Scale; NIHSS, National Institutes of Health Stroke Scale; OTD, onset to door; OTP, onset to puncture; OTR, onset to recanalization; PTR, puncture to recanalization; SBP, systolic blood pressure; SD, standard deviation; sICH, symptomatic intracerebral hemorrhage.

^a^ Analysed by Student's *t*‐test.

^b^ Analysed by Chi‐square test or Fisher's exact test.

^c^ Analysed by Mann–Whitney *U* test.

### 3.2 Thrombus Metabolomic Profiles and Short‐Term and Long‐Term Outcomes

**FIGURE 1:**
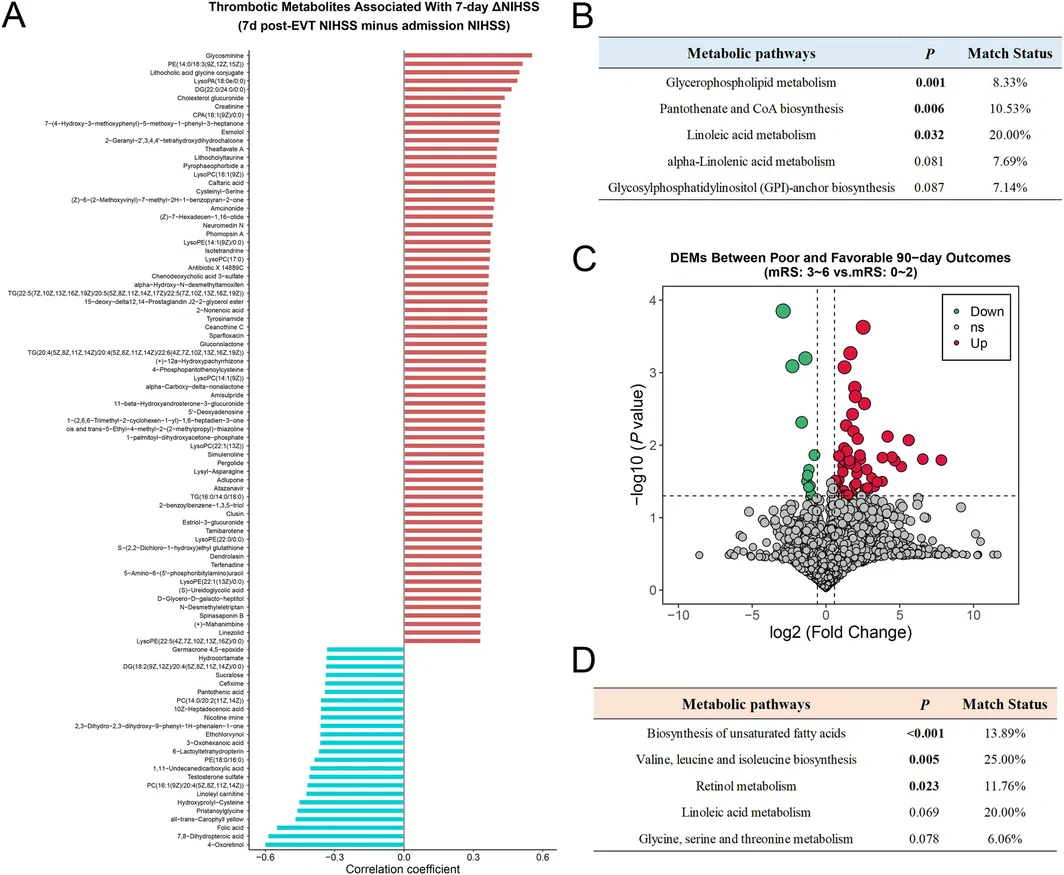
Identification of thrombus metabolites relevant to short‐term and long‐term outcomes. (A) Ninety‐four thrombus metabolites (70 positive and 24 negative) significantly correlated with 7‐day ∆NIHSS. (B) KEGG metabolic pathways enriched among the 94 metabolites associated with 7‐day ∆NIHSS. (C) Volcano plot depicting 69 DEMs (56 upregulated and 13 downregulated) between patients with favorable (mRS 0–2) and unfavorable (mRS 3–6) 90‐day outcomes. (D) KEGG metabolic pathways enriched among the 69 DEMs associated with 90‐day outcomes. 7‐day ∆NIHSS, change in NIHSS (National Institutes of Health Stroke Scale) from admission to 7‐day after thrombectomy; DEM, differentially expressed metabolites; mRS, modified Rankin Scale.

After quality control, 6700 classically annotated and abundantly expressed metabolites were detected within cerebral thromboemboli, comprising 3049 and 3651 metabolites in positive and negative modes, respectively. Spearman correlation analysis identified 94 metabolites (70 positive and 24 negative) significantly associated with 7‐day ΔNIHSS (Figure 1A). KEGG analysis showed significant enrichment in glycerophospholipid metabolism, pantothenate and CoA biosynthesis, and linoleic acid metabolism (Figure 1B). Comparative analysis using PLS‐DA model and Welch's *t*‐test identified 69 DEMs (56 upregulated and 13 downregulated) between patients with favorable and unfavorable 90‐day functional outcomes (Figure 1C). These were predominantly involved in the biosynthesis of unsaturated fatty acids, valine, leucine, and isoleucine biosynthesis, and retinol metabolism (Figure 1D).

### 3.3 Unsupervised Hierarchical Clustering Analysis of Thrombus Metabolic Molecular Subtypes

**FIGURE 2:**
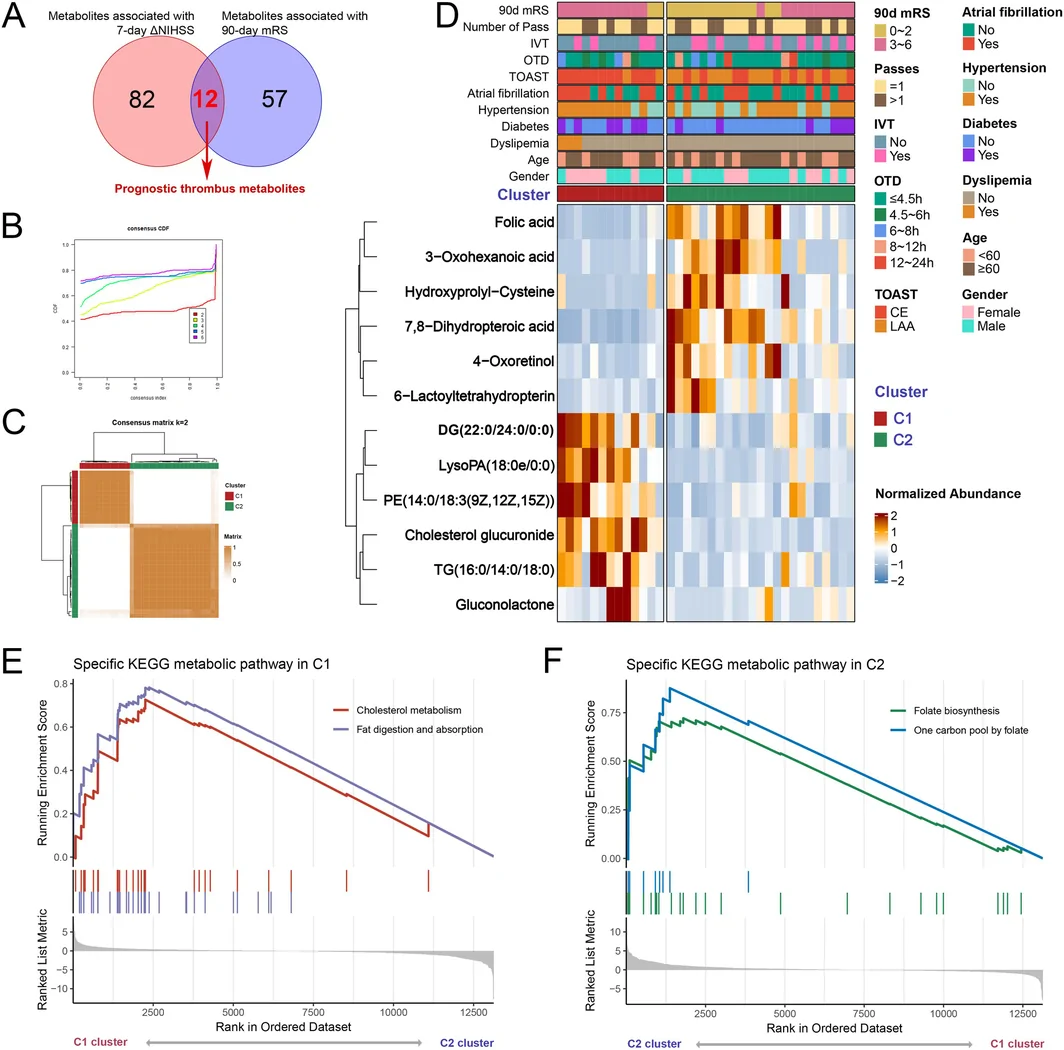
Unsupervised hierarchical clustering analysis of thrombus metabolic subtypes (A) Venn diagram illustrating the intersection of the 94 metabolites (linked to 7‐day ∆NIHSS) and 69 metabolites (linked to 90‐day mRS), yielding 12 candidate prognostic thrombus metabolites. (B) CDF curves of the consensus matrix for each cluster number *k* (*k* = 2–6) cluster. (C) Consensus score matrix for all samples with *k* = 2. (D) Global landscape of clinical characteristics and the abundance of 12 prognostic thrombus metabolites in the two metabolic subtypes. (E, F) MESA showing dominantly activated metabolic pathways in C1 (E) and C2 (F) subtypes, respectively. 7‐day ∆NIHSS, change in NIHSS (National Institutes of Health Stroke Scale) from admission to 7‐day after thrombectomy; CE, cardioembolism; IVT, intravenous thrombolysis; LAA, large artery atherosclerosis; mRS, modified Rankin Scale; OTD, onset to door; TG, triglyceride; TOAST, Trial of Org 10,172 in Acute Stroke Treatment.

Intersection of these metabolic signatures (Figure 1A,C) yielded 12 consensus metabolites linked to both short‐term and long‐term outcomes, defined as prognostic thrombus metabolites (Figure 2A). Detailed metabolite identification confidences are summarized in Table S2. Hierarchical clustering of their expression profiles categorized the 36 thromboemboli into *k* clusters (*k* = 2–6; Figure 2B). The optimal separation at *k* = 2, determined by the CDF curve and simplified classification criteria, established two metabolic molecular subtypes: Cluster 1 (C1, *n* = 13) and Cluster 2 (C2, *n* = 23) (Figure 2B,C). As illustrated in Figure 2D, C1 thromboemboli exhibited elevated levels of lipid metabolites, such as triglyceride [TG (16:0/14:0/18:0)], diglyceride [DG (20:2/24:0/0:0)], phosphoethanolamine [PE (14:0/18:3 (9Z, 12Z, 15Z))], and cholesterol glucuronide, while C2 thromboemboli were characterized by higher levels of folic acid, 7,8‐dihydropteroic acid, 6‐lactoyltetrahydropterin, hydroxyprolyl‐cysteine, 3‐oxohexanoic acid, and 4‐oxoretinol. Subsequent MSEA analysis revealed that “cholesterol metabolism” (NES = 1.85; FDR = 0.026) and “fat digestion and absorption” (NES = 1.93; FDR = 0.003) pathways were more active in the C1 subtype (Figure 2E), whereas the “folate biosynthesis” (NES = 1.63; FDR = 0.003) and “one carbon pool by folate” (NES = 1.48; FDR = 0.006) pathways were more active in the C2 subtype (Figure 2F). Based on these findings, we designated C1 as a “lipid metabolism‐enriched” thrombus subtype and C2 as a “folate metabolism‐enriched” subtype.

### 3.4 Thrombus Metabolic Molecular Subtypes and Severity, Etiology, and Prognosis

**FIGURE 3:**
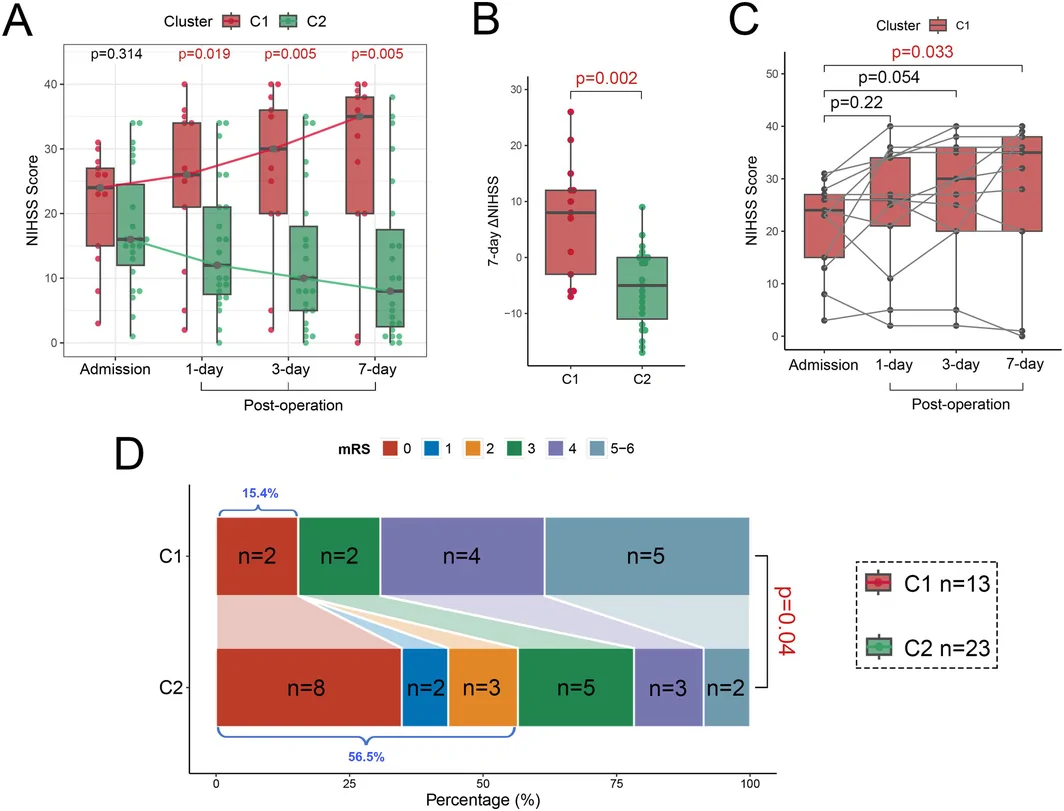
Association of thrombus metabolic molecular subtypes with short‐term and long‐term functional outcomes. (A) Comparisons of NIHSS scores at admission, and on Days 1, 3, and 7 post‐thrombectomy between the C1 and C2 subtypes. Data were presented as median (IQR) and were analyzed by Wilcoxon rank‐sum test. (B) Comparisons of 7‐day ∆NIHSS between C1 and C2. Data were presented as median (IQR) and were analyzed by Wilcoxon rank‐sum test. (C) Analysis of perioperative NIHSS changes in the C1 subtype. Data were presented as median (IQR) and were analyzed by paired Wilcoxon signed‐rank test, with *p*‐values adjusted by the Benjamini‐Hochberg FDR correction. (D) Stacked bar plot representing the distribution of 90‐day mRS. For dichotomized outcomes (favorable vs. unfavorable), group differences were analyzed using the Fisher's exact test. 7‐day ∆NIHSS, change in NIHSS (National Institutes of Health Stroke Scale) from admission to 7‐day after thrombectomy; mRS, modified Rankin Scale. Sample size: C1 (*n* = 13) and C2 (*n* = 23).

**FIGURE 4:**
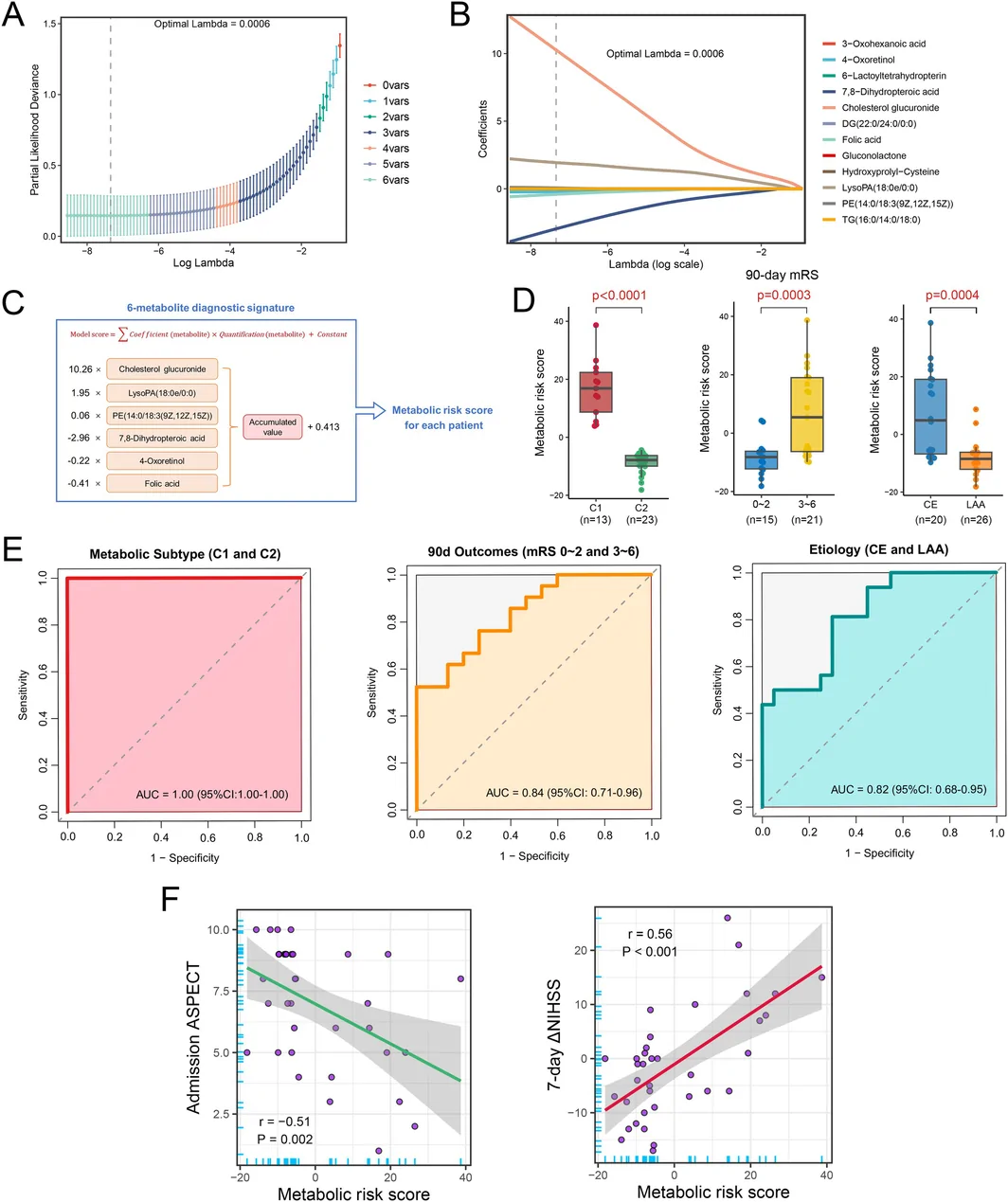
A six‐metabolite diagnostic signature for the metabolic subtypes. (A) Selection of the optimal lambda (λ = 0.0006) in LASSO regression, determined at the point of minimum partial likelihood deviance. (B) LASSO coefficient profiles of candidate metabolites used to construct the six‐metabolite model. (C) Calculation of a metabolic risk score for each patient based on the relative expression of the six metabolites, weighted by their respective LASSO regression coefficients. (D) Comparison of model‐derived risk scores across different metabolic subtypes, 90‐day outcomes, and stroke etiologies. Data were presented as median (IQR) and were analyzed by Wilcoxon rank‐sum test. (E) ROC curve analysis evaluating the diagnostic accuracy of the six‐metabolite signature for metabolic subtypes and its predictive value for 90‐day outcomes and stroke etiology. (F) Spearman correlation analysis between the six‐metabolite signature and admission ASPECTS and 7‐day ∆NIHSS. 7‐day ∆NIHSS, change in NIHSS (National Institutes of Health Stroke Scale) from admission to 7‐day after thrombectomy; ASPECTS, Alberta Stroke Program Early CT Score; CE, cardioembolism; LAA, large artery atherosclerosis; mRS, modified Rankin Scale.

Patients with C1 thrombus subtype had a higher prevalence of dyslipidemia and elevated admission diastolic blood pressure compared with C2 counterparts (Table 1). Radiographically, C1 patients presented with more severe stroke, as indicated by lower admission ASPECTS scores (Table 1). Etiologically, C1 patients were more prone to CE stroke (84.6%), whereas C2 patients more frequently experienced stroke due to LAA (60.9%) (Table 1). Among the CE cases, the distribution of underlying cardiac disorders did not differ significantly between the two subtypes (Table S1). Despite having comparable admission NIHSS scores, C2 patients exhibited significantly lower NIHSS scores on Days 1, 3, and 7 post‐EVT (Figure 3A). There was also a significant difference in the 7‐day ΔNIHSS between the subtypes, with C2 exhibiting neurological improvement reflected by a negative median 7‐day ΔNIHSS of −5 (IQR, −11–0), while C1 displayed a positive median value of 8 (IQR, −3 − 12) (Figure 3B). Notably, C1 patients developed progressive neurological deterioration, and by Day 7 post‐EVT, the NIHSS scores were significantly higher than their admission levels (Figure 3C). Furthermore, a larger proportion of C2 patients (13 out of 23 cases) achieved 90‐day neurological independence compared to C1 patients (2 out of 13 cases) (Figure 4D). These findings suggest that patients with C2 thrombus metabolic subtype experienced improved short‐term and long‐term neurological outcomes.

### 3.5 A Six‐Metabolite Diagnostic Signature for Thrombus Metabolic Subtypes

LASSO regression analysis, at an optimal λ value of 0.0006 (Figure 4A), was employed to develop a diagnostic signature distinguishing the two thrombus metabolic subtypes. This signature ultimately incorporated six metabolite variables (Figure 4B), of which five were annotated at MSI Level 2 and one (folic acid) at Level 3 (Table S2). For each patient, a metabolic risk score was calculated based on the relative expression levels of these six metabolites, weighted by their respective LASSO regression coefficients (Figure 4C). Significant differences in metabolic risk scores were observed between the thrombus metabolic subtypes (C1 vs. C2), 90‐day functional outcomes (mRS 0–2 vs. 3–6), and stroke etiologies (CE vs. LAA) (Figure 4D). The six‐metabolite signature initially achieved complete discrimination between the C1 and C2 subtypes, with an AUC of 1.00 (95% CI: 1.00–1.00) (Figure 4E). To address potential overfitting due to the limited sample size and the circular nature of the dataset, we performed rigorous internal validation. A nested 5 × 4‐fold cross‐validation scheme repeated 100 times yielded a median AUC of 1.00 (95% CI: 0.87–1.000). Consistency was confirmed through 1000 bootstrap iterations, producing a median AUC of 1.000 (95% CI: 0.9–1.000). Permutation testing indicated that the model performance was significantly better than chance (*p* = 0.013). Model calibration assessment revealed a slight deviation in the calibration curve; however, after Platt scaling, the Brier score was 0.048, suggesting acceptable calibration error. Feature selection stability was supported by the consistent inclusion of all six metabolites in > 70% of 500 internal resampling cycles, with a mean Jaccard index of 0.63. ROC curve analysis also revealed the predictive value of the six‐metabolite signature for 90‐day functional outcomes and stroke etiology, with AUC values of 0.84 (95% CI, 0.71–0.96) and 0.82 (95% CI, 0.68–0.95), respectively (Figure 4E). Additionally, the metabolic risk score was found to be positively correlated with admission infarct extension (as indicated by ASPECTS; *r* = −0.51, *p* = 0.002) and 7‐day ∆NIHSS (*r* = 0.56, *p* < 0.001), suggesting its potential as an indicator of ischemic lesion severity and short‐term neurological outcomes (Figure 4F).

## 4 Discussion

In this exploratory study designed to characterize thrombus metabolism‐based molecular subtyping for prognostic risk stratification, we initially identified 12 thrombus metabolites as candidate prognostic indicators associated with both short‐term and long‐term functional outcomes in patients with anterior circulation AIS‐LVO. For the first time, through unsupervised hierarchical clustering, we further identified two metabolically distinct thrombus molecular subtypes: a lipid metabolism‐enriched cluster (C1) and a folate metabolism‐enriched cluster (C2). Patients with C1 thromboemboli exhibited more severe ischemic lesions at admission and experienced poorer short‐ and long‐term functional outcomes compared with C2 counterparts. Finally, we established a six‐metabolite signature that demonstrated exploratory potential for discriminating thrombus metabolic molecular subtypes, etiological subtypes, and 90‐day functional outcomes, although its clinical utility warrants independent external validation.

Advancements in omics technologies and molecular subtyping have provided valuable insights into disease mechanisms and facilitated precision medicine through improved risk stratification [29]. Santo et al. [30] identified a distinct thrombus gene expression pattern associated with early neurological improvement and long‐term outcomes. Proteomic analyses by Lopez‐Pedrera et al. [31] revealed how thrombus composition and associated innate immune activity correlate with stroke etiology, severity, and progression. However, studies focusing specifically on thrombus‐specific metabolic molecular subtypes and their clinical relevance in AIS‐LVO patients remain limited. Suissa et al. [32] demonstrated the diagnostic superiority of combined thrombus proteomic‐metabolomic signatures over conventional risk factor‐based models in determining cardioembolic stroke origin. We have previously reported the thrombus metabolomic landscapes and established a correlation between thrombus metabolic profiles and stroke etiologies [16]. Another study by Suissa et al. [17] identified candidate prognostic metabolites (sorbitol and glucose) within retrieved thromboemboli, though these individual metabolites may have limited predicted specificity [29]. In this study, we identified two metabolically distinct thrombus molecular subtypes, which is a novel finding in the field of AIS‐LVO. MSEA analysis revealed that C1 thrombus subtype exhibited enhanced lipid metabolic signatures but downregulated folate biosynthesis pathways compared to C2 subtype. Notably, the etiological divergence observed between thrombus subtypes challenges prevailing paradigms. Counterintuitively, lipid metabolism‐enriched C1 subtype, despite expected associations with dyslipidemia, demonstrated predominant cardiac origin (11 out of 13 cases). A plausible explanation is that cholesterol accumulation has been mechanistically linked to endothelial dysfunction and platelet activation [33, 34], features associated with cardiac thromboemboli, which typically contain higher platelet content compared to atherosclerotic thromboemboli [2]. Our findings align with emerging lipidomic evidence demonstrating elevated TG and DG species in CE versus LAA‐related thromboemboli [20]. Although both studies are constrained by limited sample sizes necessitating further confirmation, these consistent observations suggest previously underrecognized pathophysiological heterogeneity in thromboemboli of cardiac origin. Additionally, variations in EVT treatment course, individual patient factors, and sample collection criteria may all contribute to the observed patterns. The potential mechanisms underlying metabolic‐etiologic relationships merit further investigation. An alternative interpretation also merits consideration: the higher hyperlipidemia prevalence in C1 patients may enhance intra‐thrombus lipid metabolic pathways, potentially rendering this thrombus metabolic signature an epiphenomenon.

Prognostic prediction is clinically critical for identifying suitable EVT candidates and guiding targeted adjuvant therapies [35]. In this study, patients with C1 thromboemboli frequently experienced early neurological deterioration, reflected by increasing NIHSS scores and negative 7‐day ΔNIHSS values, with only 15.4% (2/13) achieving 90‐day functional independence. This is likely attributable to the significantly higher prevalence of cardioembolic etiology in C1 patients (84.6% vs. 39.1%), a well‐established predictor of poor prognosis [6, 21]. Our findings are conceptually supported by extensive literature linking hyperlipidemia as well as folic acid deficiency and resultant hyperhomocysteinemia to both AIS pathogenesis and adverse outcomes [36, 37, 38, 39, 40]. Several mechanisms may explain these associations. Lipid accumulation potentiates thromboinflammatory cascades, blood–brain barrier disruption, and brain edema, while simultaneously aggravating systemic inflammatory responses through activating splenic and circulating neutrophils and macrophages [41, 42, 43]. These intertwined processes may collectively contribute to the no‐reflow phenomenon, secondary injury, and worse outcomes in AIS‐LVO patients [44]. Meanwhile, hyperhomocysteinemia primarily drives oxidative stress and inflammation‐induced endothelial dysfunction and neuronal damage [45, 46]. Experimental studies have further elucidated pleiotropic neuroprotective effects of folate, such as attenuation of neuroinflammatory cascades, and enhancement of angiogenesis and cellular ischemic tolerance [47, 48]. Consequently, the distinct clinical trajectories between metabolic subtypes may inform therapeutic decision‐making: C2 patients represent optimal EVT candidates, whereas C1 patients require more cautious surgical decision, enhanced perioperative monitoring, and adjuvant neuroprotective strategies despite successful recanalization. In addition, intensive lipid regulation and folate supplementation represent promising adjuvant treatments for EVT‐treated C1 subtype patients, a hypothesis that merits investigation in future multi‐center clinical trials [49, 50, 51, 52].

To enhance the molecular diagnostic approach for thrombus metabolic molecular subtypes, we developed a six‐metabolite signature using the LASSO regression, a classical machine learning algorithm [35]. In this exploratory analysis, the model showed potential for discrimination of thrombus metabolic subtypes, etiological subtypes, and 90‐day outcomes. However, the perfect AUC achieved must be interpreted cautiously, as the model was developed and tested within the same small, internally derived dataset, introducing potential circularity. Overfitting remains a significant concern despite rigorous internal validation. Accordingly, this six‐metabolite signature should be further confirmed in independent cohorts using independent analytical standards and targeted quantitative methods in future studies [25]. Notably, a major obstacle to the clinical adoption of the six‐metabolite signature is the absence of a rapid detection platform. Therefore, creating a streamlined, fast‐turnaround assay for thrombus analysis represents a key translational goal. Furthermore, to advance toward non‐invasive monitoring, it would be valuable to correlate thrombus metabolite levels with paired plasma/serum samples. Investigating corresponding changes in peripheral blood metabolites, or even routine laboratory parameters related to lipid metabolism (such as traditional and non‐traditional lipid markers) and folate pathway components (e.g., folate and homocysteine), may help identify circulating surrogates. These markers could eventually support in vitro diagnosis and facilitate clinical translation.

Our study has several limitations that affect the generalizability of our findings. First, the unsupervised clustering was performed using the 12 metabolites that were preselected based on their associations with both short‐term and long‐term outcomes. This workflow introduces an inherent bias, as prognostic information is embedded into the clustering process, which may artificially inflate the observed subtype‐outcome associations. While this pre‐filtering strategy was deliberately chosen to reduce noise from the high‐dimensional metabolomic data and to prioritize clinically relevant signals in this exploratory setting, we acknowledge this potential circularity. Future investigations should validate the clustering in independent cohorts or employ etiology‐driven metabolites to define subtypes independently of outcome measures. Second, the limited sample size constrains statistical power and increases the risk of both Type II errors and overfitting. Despite stringent internal validation, the metabolite‐to‐sample ratio remains a concern, and the perfect discrimination achieved by the six‐metabolite signature in this discovery cohort is likely overoptimistic. Thus, all findings should be interpreted as hypothesis‐generating, and external validation in larger, independent cohorts is essential. Third, all participants were of Han Chinese ethnicity. Given that dietary patterns and population‐specific metabolic backgrounds are known to influence systemic and local metabolomic profiles [9], this may limit the direct applicability of our results to other ethnic groups and geographic regions. Additionally, the potential influence of sex on thrombus metabolic profiles and clinical outcomes warrants further investigation. Fourth, the associations between thrombus metabolic subtypes and longer‐term impact beyond 90 days remain undefined. Fifth, there is potential selection bias, as it excludes thromboemboli resistant to EVT or dissolved by IVT. Sixth, this study specifically focused on thromboemboli originating from CE and LAA sources; other etiologies like cancer‐related stroke merit increasing research attention [19, 20]. Furthermore, the impact of different cardiac disorders underlying CE stroke should also be taken into account [21]. Seventh, certain key metabolites and their metabolic pathways remain unidentified, possibly overlooking other significant metabolic changes. Finally, the correlations observed between thrombus metabolic profiles and outcomes do not necessarily indicate causation, warranting further investigation. Overall, our study serves as a pilot and exploratory investigation that lays the foundation for larger, adequately powered, and prospective multicenter trials. To systematically address the issues of generalizability and standardization, we propose a validation framework that includes: (1) standardization of pre‐analytical protocols for thrombus collection, processing, and storage [53]; (2) enrollment of consecutive patients across multiple centers to capture a wide range of geographic, ethnic, and dietary backgrounds; and (3) systematic acquisition of clinical, imaging, and procedural data to adjust for center‐specific workflows, potential confounders, and transfer‐related biases.

## 5 Conclusions

In this preliminary exploratory study, we propose a metabolism‐based thrombus molecular subtyping framework that distinguishes AIS‐LVO patients with different etiologies, severities, and prognostic trajectories. The observed associations between the two metabolic subtypes and clinical outcomes generate the hypothesis that thrombus metabolic profiles may inform future risk stratification and personalized management approaches. However, further validation through multi‐center, prospective, independent cohort studies is required, along with verification using peripheral blood for in vitro diagnosis. Future research should also focus on developing a comprehensive subtyping strategy that integrates multiple omics approaches, including radiomics models [8, 54]. Additionally, our findings indicate that intensive blood lipid regulation and folic acid supplementation could be effective adjuvant treatments post‐EVT intervention.

## Author Contributions

X.X. and L.J. conceived and designed the study. X.X. and T.L. developed methodology. T.L. and W.L. carried out the thrombus metabolomics analysis. T.L., W.C., Y.L., J.W., and D.W. collected data, performed data analysis, and prepared the figures and tables. X.X. and T.L. interpreted the results and wrote the manuscript. X.X. and L.J. reviewed and revised the manuscript and supervised the study. All authors read and approved the final manuscript.

## Funding

This study was supported by grants from the National Natural Science Foundation of China (grant 82471331 and 82171303), the Taishan Scholar Youth Expert Program of Shandong Province (grant tsqn202408398), the Beijing Natural Science Foundation (grant L2510116), the Xuanwu Hospital Talent Convergence Program (grant HZ2025PYYX002), and the Wu jieping medical foundation (grant 320.6750.2024‐23‐23).

## Ethics Statement

The Ethics Committee of the Xuanwu Hospital, Capital Medical University approved this study (ID‐CRB‐61/2023), and it was conducted in accordance with the Principles of the Declaration of Helsinki.

## Consent

Written informed consent was obtained from all participants or their legal representatives.

## Conflicts of Interest

The authors declare no conflicts of interest.

## Supporting information


**Table S1:** Cardiac disorders responsible for cardioembolic stroke.
**Table S2:** Detailed information on the 12 identified metabolites.

## Data Availability

The raw data that support the findings of the current study are available from the corresponding author upon reasonable request.
