# Supplementary material for: Thrombus Metabolism‐Based Molecular Subtyping for Prognostic Risk Stratification in Acute Ischemic Stroke: A Preliminary Study
Source: CNS Neurosci Ther. 2026 Aug 30;32(9):e71116. doi: 10.1002/cns.71116 (PMC13526402; doi:10.1002/cns.71116)
Supplement: Supplementary file 1 [file CNS-32-e71116-s001.docx]

**Supplementary data**

**Thrombus metabolism-based molecular subtyping for prognostic risk stratification in acute ischemic stroke: a preliminary study**

Taoyuan Lu^1, 2, 3, †^; Wei Li^3, †^; Wenbo Cao^1, 2^; Yiying Liu^4^; Jinzi Wei^3^; Dechao Wang^1, 2^; Liqun Jiao^1, 2, 3, 5, *^; Xin Xu^1, 2, 3, *^

^1^Department of Neurosurgery, Xuanwu Hospital, Capital Medical University, 45 Changchun Street, Beijing, China

^2^China International Neuroscience Institute (China-INI), 45 Changchun Street, Beijing, China

^3^Xuanwu Hospital Jinan Branch, 5106 Jingshi Road, Jinan, Shandong, China

^4^Department of Rehabilitation Medicine, The First Affiliated Hospital of Zhengzhou University, 1 Jianshe Dong Road, Zhengzhou, Henan, China

^5^Department of Interventional Neuroradiology, Xuanwu Hospital, Capital Medical University, 45 Changchun Street, Beijing, China

**Supplementary table 1. Cardiac disorders responsible for cardioembolic stroke**

|  | **AIS Patients (n=20)** | **Thrombus Metabolic Molecular Subtypes** | | |
| --- | --- | --- | --- | --- |
|  |  | **C1 (n=11)** | **C2 (n=9)** | ***p value*** |
| Cardiac disorders for cardioembolic stroke, n (%) |  |  |  |  |
| Atrial fibrillation | 16 (80.0) | 8 (72.7) | 8 (88.9) | 1.000^a^ |
| Patent foramen ovale  foramen ovale | 2 (10.0) | 1 (9.1) | 1 (11.1) |  |
| Rheumatic heart disease | 1 (5.0) | 1 (9.1) | 0 (0.0) |  |
| Endocarditis | 1 (5.0) | 1 (9.1) | 0 (0.0) |  |

Note: ^a^Analysed by Fisher's exact test

**Supplementary table 2. Detailed information on the 12 identified metabolites**

| **Description** | **Compound ID** | **M/Z** | **Retention time (min)** | **Adduct type** | **Annotation confidence (Score)** | **Fragmentation Score** | **Mass Error (ppm)** | **Isotope Similarity** | **MSI**  **level** |
| --- | --- | --- | --- | --- | --- | --- | --- | --- | --- |
| 4-Oxoretinol | HMDB0012329 | 339.1717447 | 5.359083333 | M+K | 38.2 | 5.2 | -1.14422641 | 92.38504886 | 2 |
| 7,8-Dihydropteroic acid | HMDB0001412 | 332.1463408 | 3.72065 | M+NH4 | 39.4 | 9.98 | -0.710063516 | 87.72952957 | 2 |
| Cholesterol glucuronide | HMDB0010330 | 561.3796611 | 2.1773 | M-H | 39.2 | 3.29 | -0.029359677 | 96.2767623 | 2 |
| Folic acid | HMDB0000121 | 422.1202672 | 1.657533333 | M-H2O-H | 36.3 | 6.8 | -3.565932728 | 85.46020371 | 3 |
| LysoPA(18:0e/0:0) | HMDB0011144 | 463.2583279 | 4.963966667 | M+K | 36.2 | 10.6 | -0.485402397 | 70.79966392 | 2 |
| PE(14:0/18:3(9Z,12Z,15Z)) | HMDB0008831 | 703.4981639 | 4.739016667 | M+NH4 | 31.7 | 3.15 | -5.713450224 | 65.13291 | 2 |
| 3-Oxohexanoic acid | HMDB0010717 | 148.0969656 | 4.914516667 | M+NH4 | 38.3 | 4.6 | 1.12174305 | 93.00092682 | 2 |
| Hydroxyprolyl-Cysteine | HMDB0028860 | 252.1011382 | 0.913483333 | M+NH4 | 38.6 | 0 | -0.492293311 | 93.42833942 | 4 |
| Gluconolactone | HMDB0000150 | 177.0394366 | 5.394533333 | M-H | 37.2 | 0 | -5.756742929 | 92.36654475 | 4 |
| DG(22:0/24:0/0:0) | HMDB0007615 | 787.7205553 | 4.623416667 | M+Na | 39.4 | 9.69 | 7.26853481 | 95.68258182 | 2 |
| TG(16:0/14:0/18:0) | HMDB0010413 | 829.7325804 | 4.70025 | M+Na | 31.2 | 0 | 8.700335487 | 65.64189422 | 4 |
| 6-Lactoyltetrahydropterin | HMDB0002065 | 477.1989519 | 6.8187 | 2M-H | 37.1 | 8.1 | 5.331940221 | 83.55151635 | 3 |

Note: MSI, metabolites were structurally identified using Metabolomics Standards Initiative (MSI) confidence levels.
